# Comparison of a portable Vis-NIR hyperspectral imaging and a snapscan SWIR hyperspectral imaging for evaluation of meat authenticity

**DOI:** 10.1016/j.fochx.2023.100667

**Published:** 2023-04-03

**Authors:** Abolfazl Dashti, Judith Müller-Maatsch, Emma Roetgerink, Michiel Wijtten, Yannick Weesepoel, Hadi Parastar, Hassan Yazdanpanah

**Affiliations:** a Wageningen Food Safety Research, Wageningen University and Research, Wageningen, the Netherlands; b Forensic Toxicology Department, Legal Medicine Research Center, Legal Medicine Organization, Tehran, Iran; c Department of Toxicology and Pharmacology, School of Pharmacy, Shahid Beheshti University of Medical Sciences, Tehran, Iran; d Department of Chemistry, Sharif University of Technology, Tehran, Iran; e Food Safety Research Center, Shahid Beheshti University of Medical Sciences, Tehran, Iran

**Keywords:** Portable HSI, Snapscan HSI, Meat authenticity, PCA, Chemometrics

## Abstract

The performance of visible-near infrared hyperspectral imaging (Vis-NIR-HSI) (400–1000 nm) and shortwave infrared hyperspectral imaging (SWIR-HSI) (1116–1670 nm) combined with different classification and regression (linear and non-linear) multivariate methods were assessed for meat authentication. In Vis-NIR-HSI, total accuracies in the prediction set for SVM and ANN-BPN (the best classification models) were 96 and 94 % surpassing the performance of SWIR-HSI with 88 and 89 % accuracy, respectively. In Vis-NIR-HSI, the best-obtained coefficient of determinations for the prediction set (R^2^_p_) were 0.99, 0.88, and 0.99 with root mean square error in prediction (RMSEP) of 9, 24 and 4 (%w/w) for pork in beef, pork in lamb and pork in chicken, respectively. In SWIR-HSI, the best-obtained R^2^_p_ were 0.86, 0.77, and 0.89 with RMSEP of 16, 23 and 15 (%w/w) for pork in beef, pork in lamb and pork in chicken, respectively. The results ascertain that Vis-NIR-HSI coupled with multivariate data analysis has better performance rather than SWIR-HIS.

## Highlights

• Portable Vis-NIR and SWIR hyperspectral imaging were used for meat authenticity.
• Linear and non-linear chemometrics methods were used to analyze the data.
• The best results were achieved with Vis-NIR HSI combined with non-linear methods.
• Vis-NIR and SWIR HSI may be used for online/in-line routine screening of meat authenticity.

## 1 Introduction

Meat authenticity and meat fraud detection are challenging for scientists, industries, regulatory agencies, and consumers. Meat authenticity is, however, important regarding different social and health issues, in particular for consumers following a religious diet (Halal, kosher, etc.) and who suffer from allergies (Dashti et al., 2021; Mohammed Kamruzzaman, 2021). Also, the authenticity of food products affects the credibility of the companies. Different analytical methods including, polymerase chain reaction (PCR) (Kumar, 2021), chromatographic and mass spectrometry (MS) techniques (Stachniuk et al., 2021), and immunoassay-based methods (Mandli et al., 2018) were developed for meat authentication. Current approaches for meat fraud detection have different limitations such as being time-consuming, laborious, expensive, and destructive. In addition, they usually require high-skill technicians (Dashti et al., 2021; Mohammed Kamruzzaman, 2021; Mohammed Kamruzzaman et al., 2016). Hence, there is a need for developing new non-invasive, rapid and accurate detection techniques, especially to design online/portable detection instruments/devices that can be applied throughout the food chain (Zhao et al., 2019). Hyperspectral imaging integrates both spectroscopic and imaging techniques in one system. The combined nature of techniques provides simultaneously physical and spatial characteristics such as shape, size, appearance and color of the sample under analysis and intrinsic chemical and molecular information thereof through spectral analysis (Wu and Sun, 2013a, Wu and Sun, 2013b). Among the available technologies, hyperspectral imaging (HSI) is a relatively new technology that has provided a promising alternative, offering speed, accuracy, and reliability, besides being a nondestructive analysis technology for different production processes (Siche et al., 2016). Recently, HSI techniques have received much attention for application in non-destructive food analysis and therefore also in meat and meat products. These techniques have been successfully implemented for meat quality (Jia et al., 2022; Mohammed Kamruzzaman et al., 2013, Zhang et al., 2019), offal adulteration detection in ground meat (Grundy et al., 2022, Jiang et al., 2021; M Kamruzzaman et al., 2014) and meat speciation (Al-Sarayreh et al., 2017, Jiang et al., 2019; Mohammed Kamruzzaman et al., 2012; Mohammed Kamruzzaman et al., 2013, Rady and Adedeji, 2020). Previous studies have mainly used benchtop hyperspectral instruments for the application of meat authenticity. Considering the large amount of adulteration of meat and the need for rapid detection at various stages of the meat supply chain, there is an urgent need to develop non-destructive, fast and portable techniques for on/in site analysis of meat authenticity at various stages of meat supply chain.

The objective of this study was: (a) to compare the practicability of two hyperspectral imaging systems with different wavelength ranges including portable visible-near infrared (400–1000 nm) and a snapscan short wave infrared (SWIR, 1116–1670 nm) in combination with different linear and non-linear classification multivariate data analysis to identify authentication of different meat species (lamb, beef, chicken and pork); (b) to quantify the pork in lamb, beef and chicken samples with dereferent linear and non-linear regression methods. Finally,this research can provide best wavelength (regarding with these cameras) and multivariate methods for meat speciation behind inherent features of portable and snapscan HSI.

## 2 Material and methods

### 2.1 Collection and preparation of meat samples

Lamb (n = 40), beef (n = 40), chicken (n = 40), and pork (n = 40) meat samples (in total, 160 samples) were purchased from local butchers and supermarkets in the Netherlands over four months in 2020 (Table S1). Species-specific PCR was used as a reference method to verify the species of the samples (Scholtens et al., 2017). This method was selected due to its high sensitivity and relative rapid analyses. Samples that tested negative were removed from the dataset.

Mixtures of meat samples (approximately 300 g) were prepared by manually removing skin and visible fat with a kitchen knife, cut into small cubes (2x2x2 cm), and ground separately using a meat grinder (Tristar VM-4210, Smartwares group, Tilburg, The Netherlands) with the coarse grinding disc (hole diameter 0.7 cm). After grinding, parts of the ground meat samples were mixed by hand. The mixtures were prepared by adding 2 %, 5 %, 10 %, 25 %, and 50 % pork (w/w) to lamb, beef, and chicken samples. Homogeneity was checked visually. Mixtures were prepared by randomization of samples (Table S2). Samples were vacuum-sealed using and stored at −18.5 °C in the dark and conditioned at room temperature for 12 h in sealed containers before data acquisition.

### 2.2 Data acquisition

#### 2.2.1 Vis-NIR (400–1000 nm) spectral imaging

Data acquisition in the Vis-NIR was performed using a portable Specim IQ hyperspectral line-scan camera with push-broom technology (Specim, Spectral Imaging ltd., Oulu, Finland). Image acquisition was performed in the wavelength range of 400–1000 nm in reflectance mode. The spatial resolution of the recorded data was 512x512 pixels and the spectral resolution was 7 nm resulting in 204 spectral bands across the wavelength range. A halogen-based illumination (2 × 1250 W) that covers the full wavelength range of 400 to 1000 nm range was used. A 95 % white reference tile was used for calibration before data acquisition.

#### 2.2.2 SWIR (1116–1670 nm) spectral imaging

An IMEC SWIR hyperspectral Snapscan imaging camera (Interuniversity Microelectronics Centre, Leuven, Belgium) with 108 spectral bands and a spectral range of 1116–1670 nm equipped with an Optec 16 mm F1.7 SWIR lens (Optec S.p.A., Parabiago, Italy) was used. The system was controlled by the IMEC Snapscan software (version 1.3.0.8, IMEC). Four small halogen lamps were utilized for the homogeneous illumination of the samples. Before data acquisition, the camera was calibrated using a 95 % reflection calibration white standard (WS) tile sized 200 × 200 mm, using an integration time of 2.5 ms for the 640 × 512 × 108 (x * y * λ) hypercubes.

### 2.3 Data analysis

Background segmentation, extraction of spectral data from the hyperspectral images and calculations performed in the present research were carried out using MATLAB (R2017b, The Mathworks Inc.), PLS ToolBox (R9.0, Eigenvector Inc.), and MIA_Toolbox (version 3.1, Eigenvector Inc.). ROIs from the Vis-NIR data were selected by applying the mask. The mask was created by subtracting a low-reflectance band from a high-reflectance band.

ROIs from the SWIR images were selected by manual removal of the background using the MIA toolbox. The mean spectra of ROIs were calculated by averaging the spectra of all pixels within the ROI at each wavelength.

#### 2.3.1 Exploring the pattern in pure meat samples

Principal component analysis (PCA) was used to indicate clustering patterns of the samples (PCA score plots) and to assess wavelengths that have the most influence on the classification of samples (PCA loading plots) (Abdi & Williams, 2010).

#### 2.3.2 Discrimination (classification) of pure meat samples

For species classification of samples linear and non-linear chemometric models were tested: partial least-squares discriminant analysis (PLS-DA) (Chevallier et al., 2006), support vector machine (SVM) (Jakkula, 2006)with radial basis function and artificial neural networks based on backpropagation network (ANN-BPN) (Wythoff, 1993).

Venetian blinds cross-validation was used to assess the performance of the model and minimize the risk of overfitting data. In PLS-DA, the optimal number of PLS factors (latent variables (LVs)) for the models was selected by Venetian blinds cross-validation (number of data split: 10, thickness: 1) according to minimum RMSEC and RMSECV plots. The performance of classification models was evaluated according to the percentage of samples truly classified during calibration development and, afterward, with external validation. These performance parameters, such as sensitivity, specificity, accuracy and error rate are usually derived from a confusion matrix, to better assess the classification performance.

These parameters were calculated according to the subsequent expressions combining the number of true positives (TP, correctly identified), true negatives (TN, correctly rejected), false positives (FP, incorrectly identified) and false negatives (FN, incorrectly rejected) achieved in calibration and validation. These parameters are defined according to Equations (1), (2), (3), (4) (Ballabio and Consonni, 2013, Tharwat, 2018).

(1) $$\;Sensitivity=\frac{TP}{TP+FN}$$

(2) $$Specificity=\frac{TN}{TN+FP}$$

(3) $$Accuracy=\frac{TP+\;TN}{TP+TN+FP+FN}$$

(4) $$Error\;rate=1-Acc=\frac{FP+FN}{TP+TN+FP+FN}$$

#### 2.3.3 Regression methods

For identification of the presence of port in meat samples, different regression linear and non-linear chemometric models were tested: partial least squares (PLSR), support vector machines regressing (SVM-R) and artificial neural networks based on multi-layer back-propagation perceptron (ANN-MLP) were applied to predict the percentage of adulteration.

### 2.4 Software

Clustering (PCA), classification (PLS-DA, SVM and ANN-BPN) and regression (PLSR and SVM-R) analysis were performed using PLS-toolbox version 9.0 (Eigenvector, USA). Also, ANN-MLP regression model was applied using MVC1 toolbox (Olivieri et al., 2004) written for MATLAB. Before chemometric modeling, an appropriate data pretreatment was chosen based on the performance of the chemometric model. Effects of the different data pretreatments are displayed in the results and discussion section. A flowchart that explains the complete analysis of the hyperspectral data starting from image acquisition to multivariate analysis is shown in Figure S1.

## 3 Results and discussion

**Fig. 1:**
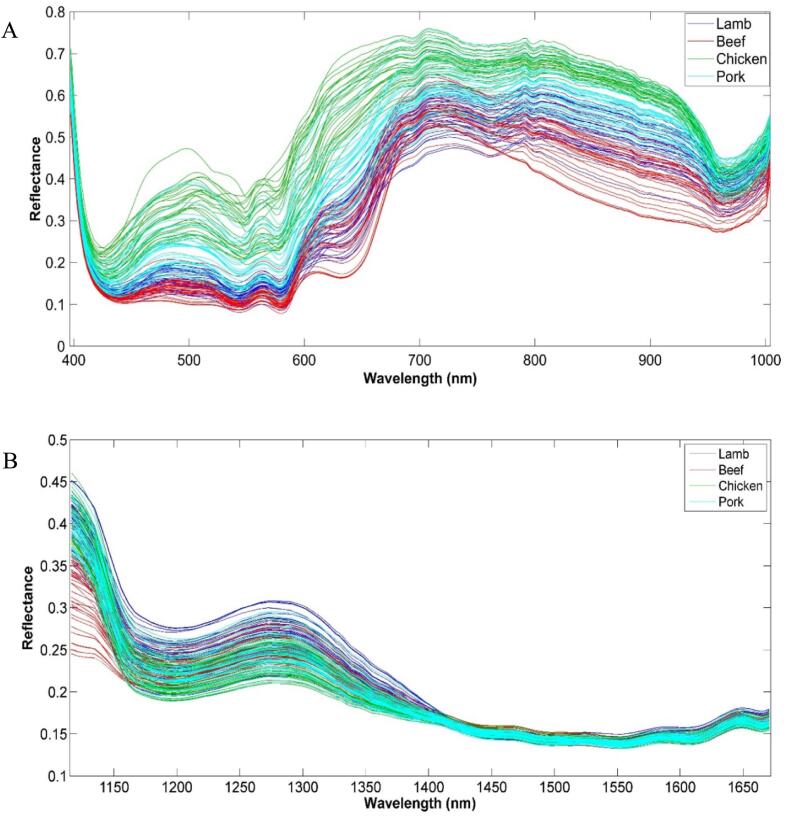
A) Vis-NIR and B) SWIR averaged spectra obtained from all four pure meat species.

Fig. 1 shows Vis-NIR and SWIR averaged spectra obtained from four pure meat species. According to the heavy overlap of the spectra for different samples, chemometric analysis is necessary for the extraction of useful information.

### 3.1 Exploring the pattern in pure meat samples

Different preprocessing techniques were tested and the Gap segment 2nd order derivative with external parameter orthogonalization (EPO) (2 PCs) and mean center data preprocessing steps were applied before PCA. These pre-processing methods were found preferable for both Vis-NIR and SWIR HSI cameras, respectively (data of other pre-processing tested not displayed).

**Fig. 2:**
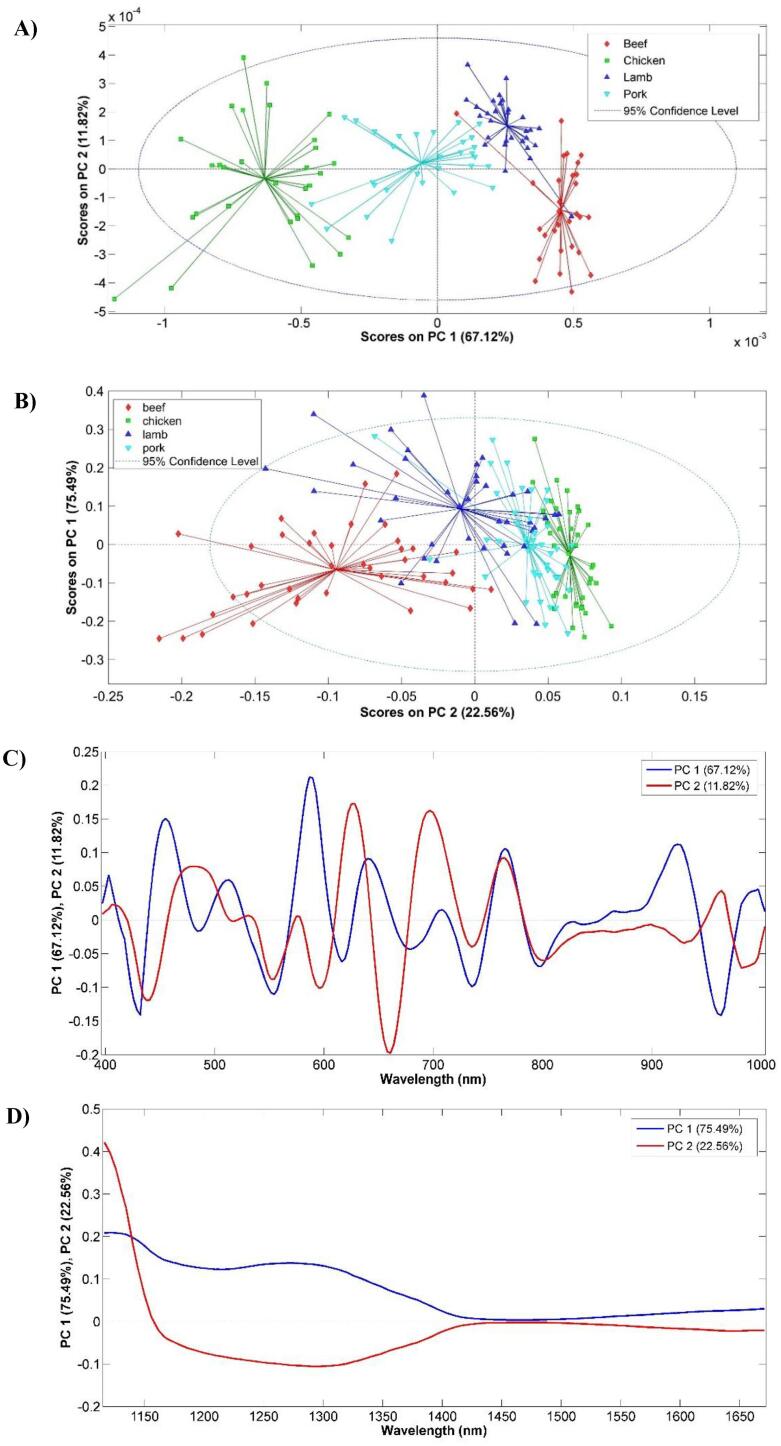
The PCA score plots of Vis-NIR (A) and SWIR (B) and PCA loading plot of Vis-NIR spectra preprocessed with Gap segment 2nd order derivative with external parameter orthogonalization (EPO) (2 PCs) (C) and SWIR spectra preprocessed with mean center (D) for pure meat samples.

According to the PCA score plots in Fig. 2 A and B, separations between groups in score space for both cameras were found when classifying the four different meat species. In the Vis-NIR PCA score plot, PC1 is corresponding to the maximum variance capture and provides the best separations (clustering) between species. The highest loading on PC1 and PC2 (Fig. 2 C) were found around 418, 550 and 578 nm associated with meat pigments and wavelength between 800 and 1000 nm could be related to meat composition (water, protein and fat,..). On contrary, PC1 is corresponding to the maximum variance but the best clustering was on PC2 in the SWIR PCA score plot. According to the loading on PC1 and PC2 (Fig. 2 D), the wavelengths between 1116 and 1500 nm (associated with the water and protein content) have more contributions to samples clustering (Cozzolino and Murray, 2004, Downey et al., 2000).

The better separation between the species in PCA score plots of Vis-NIR spectra compared with SWIR spectra may be caused by the different wavelength ranges. In the Vis-NIR features, the myoglobin and hemoglobin content of the different meat species can be incorporated into the information, while in the SWIR more information on the macro-composition like moisture, protein, and fat is detected.

Therefore, the adequate separation between species in Vis-NIR HSI rather than SWIR HSI could be related to both pigment color (visible region) and muscle composition (NIR region) information. These results are in agreement with those reported by other authors (Alomar et al., 2003, Cozzolino and Murray, 2004; Mohammed Kamruzzaman et al., 2016, Moran et al., 2018).

Interestingly, the type of cut was not found to be relevant in the PCA analysis.

Furthermore, the wavelengths with a higher value are important to explain the variance and are potential wavelengths to differentiate the four species (see Fig. 2 C and D). These wavelengths can be used for meat classification instead of the whole spectral range.

### 3.2 Discrimination (classification) of pure meat samples

The most appropriate pre-processing strategy was chosen according to the highest sensitivity, specificity, and lowest classification error. The best performing PLS-DA models for Vis-NIR and SWIR cameras were achieved with baseline correction in addition to 1st derivative (order: 2, window 19pt) and 2nd derivative (order: 2, window 21pt), and OSC (orthogonal signal correction), respectively (Table 1, Table 2).

**Table 1:** Classification performance (in %) of PLS-DA, SVM (kernel function: RBF), and ANN (BPN algorithm) models for classification of lamb, beef, chicken, and pork with Vis-NIR hyperspectral camera (400–1000 nm).

		PLS-DA^1^			SVM^2^			ANN^3^		
		Train	CV	Test	Train	CV	Test	Train	CV	Test
Lamb	Sensitivity	95.5	91.0	87.5	100.0	95.5	87.5	100.0	86.4	87.5
	Specificity	88.5	85.2	82.1	100.0	96.7	96.4	100.0	98.4	92.9
	Accuracy	92.0	88.1	84.9	100.0	96.1	92.0	100.0	92.4	90.2
	Error	8.0	11.9	15.1	0.0	3.9	8.0	0.0	7.6	9.8
										
Beef	Sensitivity	100.0	100.0	77.8	100.0	100.0	88.9	100.0	100.0	88.9
	Specificity	98.4	92.1	88.9	100.0	100.0	96.3	100.0	98.4	96.3
	Accuracy	99.2	96.1	83.4	100.0	100.0	92.6	100.0	99.3	92.6
	Error	0.8	3.9	16.6	0.0	0.0	7.4	0.0	0.7	7.4
										
Chicken	Sensitivity	100.0	100.0	100.0	100.0	100.0	100.0	100.0	100.0	100.0
	Specificity	100.0	96.7	100.0	100.0	98.3	100.0	100.0	100.0	100.0
	Accuracy	100.0	98.4	100.0	100.0	99.2	100.0	100.0	100.0	100.0
	Error	0.0	1.6	0.0	0.0	0.8	0.0	0.0	0.0	0.0
Pork	Sensitivity	83.3	72.2	91.7	100.0	83.3	100.0	100.0	61.1	91.7
	Specificity	83.3	80.0	91.7	100.0	98.5	100.0	100.0	87.7	100.0
	Accuracy	83.2	76.2	91.7	100.0	90.9	100.0	100.0	74.5	95.9
	Error	16.8	23.8	8.3	0.0	9.1	0.0	0.0	25.5	4.1

1 preprocessed with baseline correction + 1st derivative (order: 2, window 19pt).

2 preprocessed with 1st derivative (order: 2, window: 15pt).

3 preprocessed with SNV.

**Table 2:** Classification performance (in %) of PLS-DA, SVM (kernel function: RBF), and ANN (BPN algorithm) models for classification of lamb, beef, chicken, and pork with SWIR hyperspectral camera (1116–1670 nm).

		PLS-DA^1^			SVM^2^			ANN^3^		
		Train	CV	Test	Train	CV	Test	Train	CV	Test
Lamb	Sensitivity	92.6	88.9	83.3	77.8	70.4	58.3	88.9	77.8	83.3
	Specificity	65.5	58.3	47.2	97.6	90.5	91.7	91.7	78.6	86.1
	Accuracy	79.1	73.7	65.3	87.7	80.4	75.0	90.3	78.2	84.8
	Error	20.9	26.3	34.7	12.3	19.6	25.0	9.7	21.8	15.2
Beef	Sensitivity	100.0	95.5	100.0	95.5	86.4	94.1	95.5	90.9	88.2
	Specificity	94.4	88.8	100.0	100.0	100.0	96.8	96.6	93.3	93.5
	Accuracy	97.2	92.2	100.0	97.7	93.2	95.4	96.1	92.1	90.9
	Error	2.8	7.8	0.0	2.3	6.8	4.6	3.9	7.9	9.1
Chicken	Sensitivity	93.1	86.2	100.0	89.7	86.2	100.0	100.0	89.7	100.0
	Specificity	85.4	79.3	83.8	97.6	90.2	100.0	100.0	91.5	100.0
	Accuracy	89.3	82.8	91.9	93.6	88.2	100.0	100.0	90.6	100.0
	Error	10.7	17.2	8.1	6.4	11.8	0.0	0.0	9.4	0.0
Pork	Sensitivity	93.9	90.9	100.0	87.9	60.6	75.0	97.0	75.8	75.0
	Specificity	38.5	41.0	52.5	87.2	84.6	90.0	97.4	74.4	90.0
	Accuracy	66.3	66.0	76.3	87.5	72.6	82.5	96.6	75.1	82.5
	Error	33.7	34.0	23.7	12.5	27.4	17.5	3.4	24.9	17.5

1 preprocessed with 2nd derivative (order: 2, window 21pt) and OSC (orthogonal signal correction).

2 preprocessed with 2nd derivative (order: 2, window: 9pt) and Pareto (Sqrt Std) scaling.

3 preprocessed with SNV (standard normal variate).

In this regard, the best total accuracy values for Vis-NIR and SWIR were 90 % and 83 %, respectively. Also in the SVM method, the best appropriate pre-processing strategy was chosen according to the highest sensitivity, specificity, and lowest classification error. Hereof, 1st derivative (order: 2, window: 15pt) and 2nd derivative (order: 2, window: 9pt) plus Pareto scaling(scale each variable by the square root of its standard deviation (Sqrt Std) were the most proper pre-processing methods for Vis-NIR and SWIR, respectively. The best total accuracy values for Vis-NIR and SWIR were 96 % and 88 %, receptively (Table 1, Table 2). For ANN-BPN methods, the best results were achieved with SNV for both cameras and the best total accuracy was 94 % and 89 % for Vis-NIR and SWIR, respectively (Table 1, Table 2). Table 1, Table 2 show that non-linear models like SVM with RBF kernel and ANN-BPN, have a better prediction ability than the linear models (PLS-DA) for both HSI systems.

The total accuracy for Vis-NIR was arranged between 90 and 96 % and the total accuracy for SWIR was 83–89 %. In our work, the Vis-NIR camera has superior performance for pure meat classification of lamb, beef, chicken, and pork in comparison with the SWIR camera. It could be due to the different and longer wavelength range of the Vis-NIR hyperspectral camera (400–1000 nm) rather than the SWIR hyperspectral camera (1116–1670 nm). Visual differences were observed between sample species in both visible and NIR regions (Fig S1 A).

Peaks around 418, 546, and 578 nm may be related to hemoglobin and myoglobin absorption. This showed that both pigment color information (from the visible region) and the composition of the meat (from the NIR region) gave information to be used for speciation purposes (Alamprese et al., 2013, Cozzolino and Murray, 2004, Dashti et al., 2021, Moran et al., 2018). Snapscan HSI systems have limited spectral information for red-meat speciation and authentication while proposing advantages such as portability and video rate imaging (Al-Sarayreh et al., 2020).

### 3.3 Detection of adulteration levels of pork in the meat of other species

The main objective of this contribution was to quantify the level of adulteration (pork) in minced lamb, beef, and chicken. The regression models were developed using PLS, SVM, and ANN-MLP to correlate the spectral data with the level of adulteration. Here different spectral pre-treatments were assessed for all these models and the best models were selected according to the highest R^2^ and lowest RMSE for calibration and cross-validation. Full cross-validation and test set were utilized to validate internally and externally all predictive models. As illustrated in Table 3, overall, the performance of the ANN-MLP regression model (as a fully non-linear model) in the case of cross-validation and prediction was better than PLS and SVM regression models. In Vis-NIR hyperspectral camera, the best quantification models belong to the detection of pork in beef, pork in chicken, and the end, pork in lamb, respectively. The weaker results for the quantification of pork in lamb could be due to the similarity between their spectra (Fig. S2 A).

**Table 3:** Performance comparison of the three regression models for quantification of pork in beef, lamb, and chicken meat samples (without preprocessing and with the best preprocessing) with Vis-NIR hyperspectral camera (400–1000 nm).

		Preprocessing	LVs	RMSEC	RMSECV	RMSEP	R^2^_C_	R^2^_CV_	R^2^_P_
PLS-R	Pork in beef	Non	5	12	15	17	0.83	0.74	0.83
		2nd derivative	4	10	12	14	0.90	0.83	0.90
	Pork in lamb	Non	4	16	20	22	0.79	0.68	0.75
		2nd derivative	5	14	18	24	0.84	0.73	0.76
	Pork in chicken	Non	4	14	17	20	0.86	0.80	0.57
		Gap segment 2nd derivative	8	8	17	13	0.95	0.81	0.82
SVM-R	Pork in beef	Non	----	13	17	21	0.82	0.68	0.87
		EMSC	----	9	13	9	0.91	0.81	0.95
	Pork in lamb	Non	----	16	20	23	0.81	0.70	0.80
		----	----	----	----	----	----	----	----
	Pork in chicken	Non	----	12	20	17	0.90	0.74	0.73
		Baseline correction	----	7	15	10	0.97	0.83	0.86
ANN-MLP	Pork in beef	Non	----	----	6	15	----	0.98	0.98
		Detrend	----	----	6	9	----	0.98	0.99
	Pork in lamb	Non	----	-----	22	27	----	0.78	0.87
		Centering	----	-----	20	24	----	0.81	0.88
	Pork in chicken	Non	----	-----	5	10	----	0.99	0.94
		Detrend	----	-----	5	4	----	0.99	0.99

Latent variables (LVs), Root Mean Squared Error of Calibration (RMSEC), Root Mean Squared Error of Cross-Validation (RMSECV), Root Mean Squared Error of Prediction (RMSEP).

The results revealed that spectral pre-treatment methods offer more improvement in the performance of PLS and SVM models. In ANN models, spectral pre-treatment methods mainly affected RMSE rather than R square (Table 3).

As can be seen in Table 4, the performance of the ANN-MLP regression model in the case of cross-validation and prediction was better than PLS and SVM regression models. Here, different spectral preprocessing methods were tried for SWIR spectra, but unfortunately, no suitable PLS and SVM regression models could be gained with those spectra. According to Table 3, Table 4, overall, the Vis-NIR camera had a better performance for pork meat adulteration quantification in other species (lamb, beef, and chicken).

**Table 4:** Performance comparison of the three regression models for quantification of pork in beef, lamb, and chicken meat samples (without preprocessing and with the best preprocessing) with SWIR hyperspectral camera (1116–1670 nm).

		Preprocessing	LVs	RMSEC	RMSECV	RMSEP	R^2^_C_	R^2^_CV_	R^2^_P_
PLS-R	Pork in beef	Non	4	22	26	19	0.64	0.49	0.53
		1st derivative + baseline correction	6	17	23	22	0.78	0.62	0.54
	Pork in lamb	Non	2	33	34	27	0.18	0.12	0.24
		EMSC	4	27	32	29	0.43	0.21	0.19
	Pork in chicken	Non	3	27	31	29	0.30	0.12	0.48
		Gap segment 2nd derivative	8	17	27	24	0.75	0.44	0.56
SVM-R	Pork in beef	Non	----	20	24	15	0.73	0.58	0.69
		SNV	----	10	19	18	0.92	0.73	0.67
	Pork in lamb	Non	-----	24	27	22	0.59	0.44	0.54
		Pareto (Sqrt Std) scaling	-----	17	23	21	0.79	0.61	0.61
	Pork in chicken	Non	-----	40	40	36	0.01	0.64	0.02
		OSC	-----	37	37	34	0.55	0.35	0.27
ANN-MLP	Pork in beef	Non	----	----	15	16	----	0.91	0.86
		--------	----	----	----	----	----	----	----
	Pork in lamb	Non	-----	-----	24	25	-----	0.75	0.65
		MSC	-----	-----	26	23		0.72	0.77
	Pork in chicken	Non	-----	-----	25	27	----	0.75	0.55
		2nd derivative (Sav Gol)	-----	-----	14	15	----	0.92	0.89

Latent variables (LVs), Root Mean Squared Error of Calibration (RMSEC), Root Mean Squared Error of Cross-Validation (RMSECV), Root Mean Squared Error of Prediction (RMSEP).

## 4 Conclusions

This contribution was carried out to compare the potential of two HSI cameras for speciation and adulteration quantification of different meat species combined with linear and non-linear classifications (PLS-D, SVM, and ANN-BPN) and regressions (PLSR, SVM-R, and ANN-MLP) multivariate methods. In classification methods, the total accuracy for Vis-NIR was between 90 and 96 % and the total accuracy for SWIR was 83–89 %. In this approach, the best performances were acquired with ANN-BPN in both HSI cameras.

For pork quantification in other species, the ANN-MLP regression method showed better performance in terms of cross-validation and prediction in both HSI systems rather than PLS and SVM regressions methods, and the best performance was acquired with the portable Vis-NIR HSI camera. The results demonstrated that the spectral data collected from portable Vis-NIR hyperspectral imaging combined with non-linear multivariate methods provide better performance for meat speciation and detection of pork adulteration in minced lamb, beef, and chicken rather than snapscan SWIR hyperspectral imaging.

**Funding**

This work was supported by the Dutch Ministry of Agriculture, Nature and Food Quality (Knowledge base grant, KB-38–001-008).

## Declaration of Competing Interest

The authors declare that they have no known competing financial interests or personal relationships that could have appeared to influence the work reported in this paper.

## Data Availability

The authors do not have permission to share data.
