# Supplementary material for: Comparison of a portable Vis-NIR hyperspectral imaging and a snapscan SWIR hyperspectral imaging for evaluation of meat authenticity
Source: Food Chem X. 2023 Apr 3;18:100667. doi: 10.1016/j.fochx.2023.100667 (PMC10314175; doi:10.1016/j.fochx.2023.100667)
Supplement: Supplementary data 1 [file mmc1.docx]

**Table S1:** Meat samples and mixtures

| Sample type | Species | Meat type / Cut | Number of pure samples | Number of mixtures |
| --- | --- | --- | --- | --- |
| Test | Lamb  (*Ovis aries*) | Leg | 20 | 19 |
|  |  | Shoulder | 20 | 19 |
|  | Beef  (*Bos taurus*) | Flank steak | 20 | 20 |
|  |  | Chuck roast | 20 | 20 |
|  | Chicken  (*Gallus gallus domesticus*) | Breast | 20 | 20 |
|  |  | Thighs | 20 | 19 |
|  | Pork  (*Sus scrofa domesticus*) | Ham | 20 | -^1^ |
|  |  | Blade | 20 | -^1^ |

^1^ mixture consisted of pork with another species

**Table S2:** Sample mixtures prepared. Pork concentrations and body parts were randomly distributed over the experiment days.

| Day | Lamb | C pork (% w/w) - Cut | Beef | C pork (% w/w) - Cut | Chicken | C pork (% w/w) - Cut |
| --- | --- | --- | --- | --- | --- | --- |
| 1 | Leg | 2 - Shoulder | Leg | 2 - Shoulder | Breast | 2 - Shoulder |
|  | Shoulder | 25 - Leg | Shoulder | 2 - Shoulder | Drumstick | 25 - Leg |
| 2 | Leg | 5 - Shoulder | Shoulder | 10 - Shoulder | Breast | 5 - Shoulder |
|  | Shoulder | 10 - Leg | Shoulder | 25 - Shoulder | Drumstick | 2 - Leg |
| 3 | Shoulder | 25 - Shoulder | Leg | 25 - Leg | Breast | 2 - Leg |
|  | Shoulder | 10 - Shoulder | Shoulder | 5 - Leg | Drumstick | 2 - Leg |
| 4 | Leg | 25 - Leg | Leg | 25 - Leg | Drumstick | 5 - Shoulder |
|  | Leg | 2 - Shoulder | Leg | 50 - Shoulder | Drumstick | 5 - Shoulder |
| 5 | Shoulder | 2 - Shoulder | Leg | 10 - Shoulder | Drumstick | 5 - Leg |
|  | Leg | 10 - Shoulder | Leg | 5 - Shoulder | Drumstick | 50 - Shoulder |
| 6 | Shoulder | 5 - Shoulder | Shoulder | 50 - Shoulder | Breast | 25 - Leg |
|  | Shoulder | 10 - Leg | Leg | 10 - Leg | Breast | 25 - Leg |
| 7 | Shoulder | 50 - Shoulder | Shoulder | 50 - Leg | Breast | 2 - Shoulder |
|  | Shoulder | 5 - Leg | Leg | 50 - Leg | Drumstick | 50 - Leg |
| 8 | Leg | 2 - Leg | Leg | 50 - Leg | Breast | 5 - Shoulder |
|  | Leg | 25 - Leg | Shoulder | 10 - Leg | Breast | 50 - Shoulder |
| 9 | Shoulder | 50 - Leg | Leg | 5 - Shoulder | Breast | 2 - Leg |
|  | Leg | 10 - Leg | Shoulder | 10 - Shoulder | Drumstick | 10 - Leg |
| 10 | Shoulder | 10 - Shoulder | Leg | 50 - Shoulder | Drumstick | 10 - Shoulder |
|  | Leg | 50 - Leg | Leg | 2 - Shoulder | Breast | 10 - Leg |
| 11 | Shoulder | 50 - Leg | Shoulder | 5 - Shoulder | Drumstick | 25 - Shoulder |
|  | Shoulder | 2 - Leg | Shoulder | 5 - Leg | Drumstick | 10 - Shoulder |
| 12 | Leg | 10 - Leg | Leg | 5 - Leg | Breast | 10 - Shoulder |
|  | Leg | 5 - Shoulder | Shoulder | 50 - Leg | Breast | 50 - Shoulder |
| 13 | Leg | 25 - Shoulder | Shoulder | 50 - Shoulder | Drumstick | 5 - Leg |
|  | Shoulder | 5 - Leg | Leg | 2 - Leg | Drumstick | 2 - Shoulder |
| 14 | Shoulder | 25 - Shoulder | Shoulder | 2 - Leg | Breast | 5 - Leg |
|  | Shoulder | 50 - Shoulder | Leg | 5 - Leg | Breast | 50 - Leg |
| 15 | Leg | 50 - Shoulder | Shoulder | 25 - Leg | Breast | 50 - Leg |
|  | Shoulder | 25 - Leg | Shoulder | 10 - Leg | Breast | 10 - Shoulder |
| 16 | Leg | 10 - Shoulder | Shoulder | 2 - Shoulder | Breast | 25 - Shoulder |
|  | Leg | 50 - Leg | Leg | 10 - Shoulder | Drumstick | 2 - Shoulder |
| 17 | Shoulder | 2 - Leg | Shoulder | 25 - Shoulder | Drumstick | 50 - Leg |
|  | Shoulder | 5 - Shoulder | Shoulder | 5 - Shoulder | Breast | 25 - Shoulder |
| 18 | Leg | 2 - Leg | Leg | 25 - Shoulder | Drumstick | 25 - Leg |
|  | Leg | 50 - Shoulder | Leg | 25 - Shoulder | Drumstick | 50 - Shoulder |
| 19 | Leg | 5 - Leg | Shoulder | 25 - Leg | Breast | 5 - Leg |
|  | Leg | 25 - Shoulder | Shoulder | 2 - Leg | Breast | 10 - Leg |
| 20 | Shoulder | 2 - Shoulder | Leg | 10 - Leg | Drumstick | 25 - Shoulder |
|  | Leg | 5 - Leg | Leg | 2 - Leg | Drumstick | 10 - Leg |

**Figure S1:** Scheme of hyperspectral images analysis


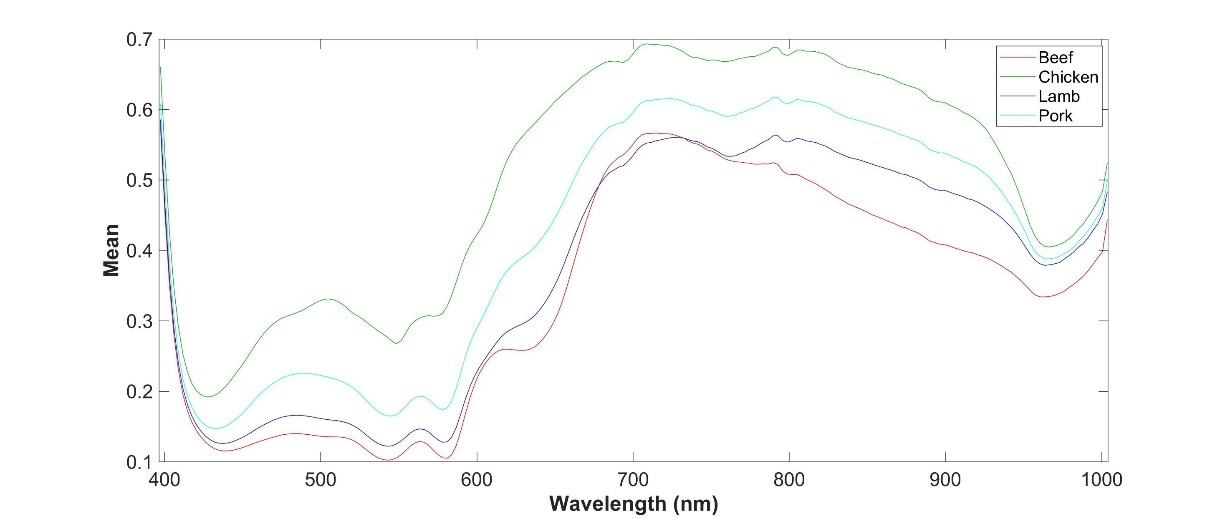

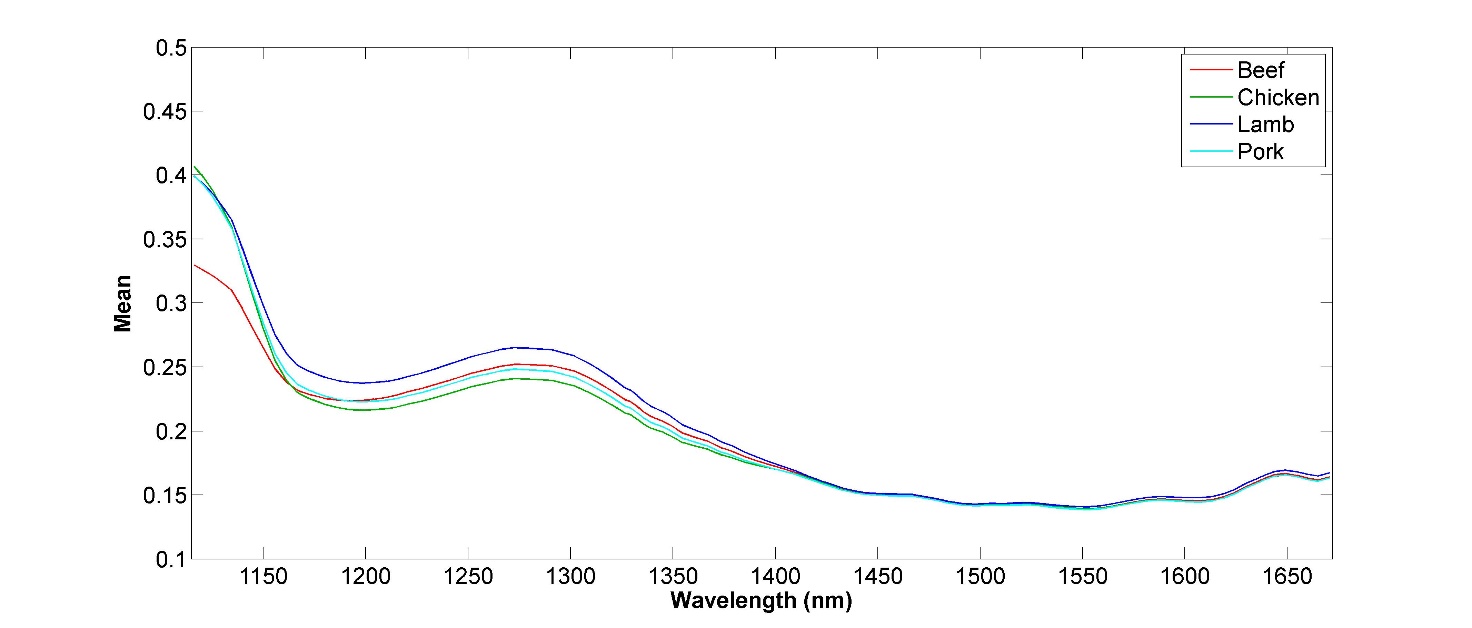


A

B

Fig S2: Mean of Vis-NIR spectra (400-1000 nm) and SWIR spectra (1116-1670 nm) of lamb, beef, chicken, and pork samples.
